# Prefrontal and Motor Planning Cortical Activity during Stepping Tasks Is Related to Task Complexity but Not Concern about Falling in Older People: A fNIRS Study

**DOI:** 10.3390/brainsci13121675

**Published:** 2023-12-05

**Authors:** Carmen Tung, Stephen Ronald Lord, Paulo Henrique Silva Pelicioni, Daina Louise Sturnieks, Jasmine Charlotte Christiane Menant

**Affiliations:** 1 Falls, Balance and Injury Research Centre, Neuroscience Research Australia, Sydney, NSW 2031, Australia; carmentung1@gmail.com (C.T.); s.lord@neura.edu.au (S.R.L.); paulo.silvapelicioni@unsw.edu.au (P.H.S.P.); d.sturnieks@neura.edu.au (D.L.S.); 2 School of Population Health, Faculty of Medicine and Health, University of New South Wales, Kensington, NSW 2052, Australia; 3 School of Health Sciences, Faculty of Medicine and Health, University of New South Wales, Kensington, NSW 2052, Australia; 4 School of Biomedical Sciences, Faculty of Medicine and Health, University of New South Wales, Kensington, NSW 2052, Australia; 5 Ageing Future Institute, University of New South Wales, Kensington, NSW 2052, Australia

**Keywords:** concern about falling, stepping, older people, cortical activity, neural efficiency, functional near-infrared spectroscopy

## Abstract

This study investigated the effect of concern about falling on neural efficiency during stepping in older people. Community-dwellers aged >65 years were categorised as having low (*n* = 71) and high (*n* = 28) concerns about falling based on the Iconographical Falls Efficacy Scale (IconFES 10-item, scores <19 and ≥19, respectively). Participants performed a choice stepping reaction time test (CSRT), an inhibitory CSRT (iCSRT), and a Stroop stepping test (SST)) on a computerised step mat. Cortical activity was recorded using functional near-infrared spectroscopy. There were no significant differences in stepping response times or cortical activity in the dorsolateral prefrontal cortex (DLPFC), supplementary motor area (SMA), and premotor cortex (PMC) between those with and without concern about falling. However, stepping response times and cortical activity in the PFC, SMA, and PMC were significantly higher in the SST compared with the CSRT in the whole sample. PMC activity was also higher in the SST compared to the iCSRT. These findings demonstrate that cortical activity is higher in cognitively demanding stepping tasks that require selective attention and inhibition in healthy older people. The lack of association between concern about falling and neural efficiency during stepping in this older sample may reflect their only moderate scores on the IconFES.

## 1. Introduction

Falls are a prominent health concern amongst older people, with around 30% of community-dwellers aged 65 years and over experiencing at least one fall per year. Falls can result in injuries requiring medical care, instill fear of falling, and decrease confidence in mobility and undertaking activities of daily living in older people [1].

Several risk factors for falls in older adults have been identified, including cognitive, balance and gait impairments, with strong evidence that these impairments are interrelated [2]. For example, it has been consistently found that age-related declines in cognitive processes, especially higher-order neural functions, are associated with gait impairment in older adults [2]. Further, it has been demonstrated that balance and gait require more cognitive control with advancing age, as documented by increased cortical activity in the prefrontal cortex (PFC) in functional neuroimaging studies [3,4]. Moreover, functional near-infrared spectroscopy (fNIRS) studies have demonstrated that cortical activity in the dorsolateral prefrontal cortex (DLPFC) is elevated during walking in older people, with larger increases evident when participants undertake secondary cognitive tasks [3,5,6,7]. These consistent findings indicate that attention and executive functions play an important role in locomotion and fall avoidance in older people [2].

Concern about falling is common among older people, affecting 20–39% of community dwellers [8]. It is present in older people who have and have not experienced a fall and can significantly reduce the quality of life [9]. There is substantial evidence that people with concern about falling exhibit behavioural changes during standing and walking, which, instead of being protective, may increase fall risk [10]. It has also been suggested that concern about falling influences attentional processes and the efficiency of the working memory [11] in that older people with a concern about falling may engage cognitive resources in task-irrelevant factors (such as ruminating on the threat of falling) that may reduce the ability to safely perform complex locomotor tasks that require increased cognitive demands [12,13]. In daily life, such concern about falling may lead to falls during walking involving additional (secondary) tasks [14].

Only two previous studies have examined cortical activity using fNIRS during walking tasks in older people with concerns about falling. Both studies found that fearful older people exhibited neural inefficiency, defined as higher DLPFC activity coinciding with similar or worse walking task performance, compared to non-fearful older people [15,16]. While these studies provide good insight into the effects of concern about falling on cortical activity, they have some limitations in that they restricted their focus to the DLPFC, and participants performed relatively unchallenging walking tasks. To date, no studies have examined cortical activity during stepping tasks in relation to concern about falling. Such tasks may be highly relevant, as they comprise composite measures of fall risk and incorporate choice and inhibitory components [17,18,19]. As voluntary stepping is less automated than walking, it likely relies on multiple brain regions, including the dorsolateral prefrontal cortex (DLPFC) [involved in executive functioning including inhibitory processes [20], the supplementary motor area (SMA) [involved in planning and generation of internally driven actions, including anticipatory postural adjustments at gait initiation [21,22,23], and the premotor cortex (PMC) [involved in sequencing of movements evoked by external stimuli [24]. 

The aim of this study, therefore, was to investigate neural efficiency in the above three cortical regions during cognitively challenging stepping tasks requiring attention, speed, and inhibition in older people with and without concern about falling, using fNIRS. Our stepping tests comprised a standard choice stepping reaction time (CSRT) test, an inhibitory choice stepping reaction time (iCSRT) test, and a Stroop stepping test (SST). All the tests required rapid decision-making and step initiation in response to a command on the computer screen, with an additional response selection component using a go–no-go test paradigm for the iCSRT and a visuo-spatial conflict resolution task for the SST.

We hypothesised that participants with high concern about falling [15] would show poorer neural efficiency (defined as higher cortical activity in the stated regions of interest (DLPFC, SMA, PMC) coinciding with similar or worse stepping task performance [15,25]) than participants with low concern about falling, when performing the more cognitively challenging stepping tasks [15]. This would be evidenced by significant interaction between concern about falling and stepping task conditions for cortical activity and/or stepping performance, whereby between-group differences would be greater in the more complex stepping tasks (in particular the SST), with the fearful participants displaying increased cortical activity alongside slower or similar stepping responses. Due to these adaptations, it was hypothesised that the group with high concern about falling would not make more errors during the stepping tasks compared to their less concerned counterparts.

## 2. Materials and Methods

### 2.1. Study Registration

We registered our hypotheses on the Open Science Framework on October 6, 2022, prior to statistical analysis of the data (https://osf.io/fypw8, accessed on 6 October 2022). 

### 2.2. Participants

The sample comprised 99 consecutive older people (71 women) who were living in Sydney, Australia, and recruited to participate in the Smart±Step randomised controlled trial of cognitive–motor interventions to prevent falls (ACTRN12616001325493) [26]. The inclusion criteria, as described in this study’s protocol [26], were as follows: (i) aged 65 years or over; (ii) English-speaking; (iii) living in the Sydney metropolitan area; (iv) independent in activities of daily living; (v) able to walk 10 m without the use of a walking aid; (vi) willing to provide informed consent and comply with this study’s protocol. Exclusion criteria included the following: an acute psychiatric condition with psychosis; an unstable medical condition that would preclude safe participation; a progressive neurological condition (e.g., Parkinson’s disease, Multiple Sclerosis, Meniere’s disease); cognitive impairment defined as a Pfeiffer Short Portable Mental Status Questionnaire score <8 [27]; residing in residential aged care; currently participating in a fall prevention trial. All participants were screened through a telephone interview. The University of New South Wales Human Research Ethics Committee approved this study, and all participants gave written consent prior to participation in the study.

### 2.3. Descriptive Measures

Participants completed a questionnaire seeking information on demographics (age, sex, and years of formal education). They undertook the Addenbrooke’s Cognitive Examination (ACER) to assess global cognition [28], the Trail Making Tests Parts A and B to assess selective attention and processing speed [29], and the Physiological Profile Assessment (a five-item battery of sensorimotor, reaction time, and balance tests from which a composite fall risk score can be derived) [30].

### 2.4. Concern about Falling

Participants completed the 10-item Iconographical Falls Efficacy Scale (IconFES), which assesses concern about falling using pictures to describe a range of activities and situations [31]. This scale contains 10 items that are scored on a four-point scale (from 1= not at all concerned to 4 = very concerned) for a total score ranging from 10 (corresponding to “no concern”) to 40 (corresponding to “extremely concerned about falling”). A validated cut-point was used to categorise participants into those with low concern about falling (scores < 19) and high concern about falling (scores ≥19) [31].

### 2.5. Experimental Protocol

**Figure 1:**
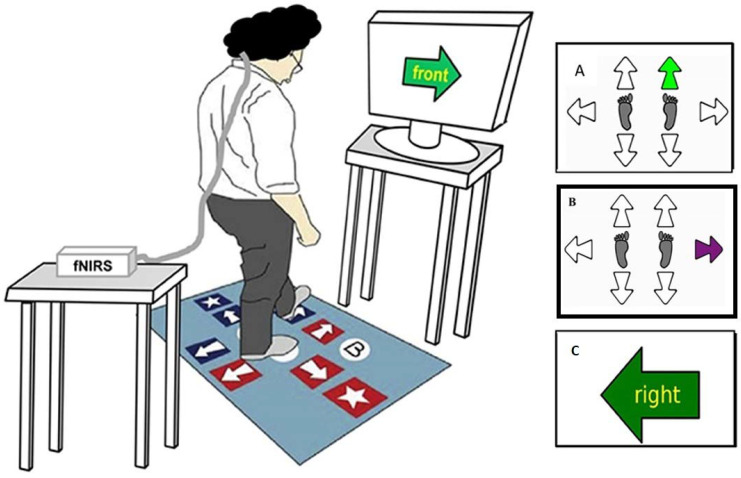
Experimental set-up. The left-handed figure represents a participant performing the Stroop stepping test (SST) on the computerised stepping mat. The right-handed figure represents the display of the following: (**A**) Choice-Stepping Reaction Time (CSRT) test, which requires participants to step as fast as possible onto the stepping mat panels corresponding to the location of the green arrow appearing; (**B**) the inhibitory choice-stepping reaction time (iCSRT) test, which requires fast and accurate stepping in response to a green arrow but inhibition of the step if the arrow is purple; (**C**) the Stroop stepping test (SST), which requires participants to step as fast as possible on the panel corresponding to the direction defined by the word in the arrow and not the orientation of the arrow itself (here, step on the right panel).

All participants performed the CSRT [17], iCSRT [18], and SST [19]. These stepping tasks were undertaken on an electronic step mat (90 × 150 cm) connected to a computer monitor (Figure 1). In brief, all the stepping tests required rapid decision-making and step initiation in response to a command on the computer screen, with the additional response inhibition and response selection (go/no-go) required for the iCSRT and the SST [19,32], respectively. In the CSRT, the participants were instructed to step onto the correct arrow according to a stimulus displayed on a screen ahead (i.e., once the arrow turns from white to green). In the iCSRT, participants were presented with the same stimulus but were told to refrain from stepping if the arrow were purple instead of green. In the SST, a large arrow was presented with a word inside (up, down, left, right), indicating a different direction, and the participant was instructed to “step by the word” as fast as possible and inhibit the response indicated by the arrow’s direction. Participants performed practice trials before each test (six for the CSRT and iCSRT, four for the SST), followed by 24, 24, and 20 randomly presented trials in the CSRT, iCSRT, and the SST, respectively. Each stepping test took approximately one minute to complete.

Performance in each stepping test was evaluated by measuring i) mean response time (mean time difference from stimulus onset to foot lifting off the computerised mat) and ii) number of errors (number of times where the foot landed on the incorrect position during each test condition).

### 2.6. fNIRS Data Acquisition and Analysis

**Figure 2:**
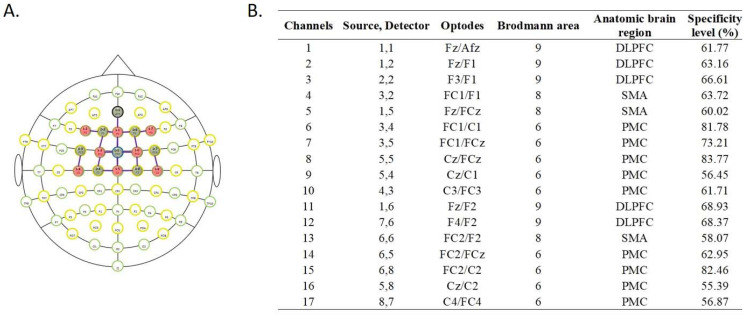
(**A**) fNIRS optodes montage (green circles: detectors; red circles: sources; purple lines: channels formed by the connection between sources and detectors). (**B**) fNIRS channels’ description, source/detector combinations, anatomic locations, and individual channel specificity (DLPFC: dorsolateral prefrontal cortex; SMA: supplementary motor area; PMC: premotor cortex). Specificity levels according to fNIRS Optodes’ Location Decider (fOLD) specifications.

Cortical activity was recorded by a continuous-wave fNIRS system (NIRSport, NIRx, Los Angeles, USA) (8 LED sources; 8 detectors; 760 nm and 850 nm frequency-modulated wavelengths; sampling rate: 7.81 Hz) while participants performed the stepping tasks. Sixteen optodes were placed on a lightweight cap based on the 10–10 international system. The fNIRS Optodes’ Location Decider (fOLD) toolbox [33] and the Brodmann area (BA) atlas were used to define the following regions of interest: DLPFC (BA 9); SMA (BA 8 or Frontal Eye Fields, which are also covered by part of the SMA); and PMC (BA 6). Optode positions, associated channels, anatomical landmarks, and their specificity are presented in Figure 2 [34]. The differential path length factor was adjusted according to participants’ age. We considered the coverage of ≥50% of a region of interest sufficient. The Cz position was considered as the reference, centred between the nasion and the inion (anteroposterior measurement) and between the left and right preauricular points (mediolateral measurement). The optodes were covered by an opaque black cap to reduce the interference of external lights. The data were recorded using NIRStar 15–2 software. Prior to each trial, calibration determined the optimal amplification factor within an optimal range (0.4–7.0 V).

Each test commenced with a brief practice (6 stepping trials for the CSRT and iCSRT tests (one in each stepping direction) and 4 stepping trials for the SST (one in each stepping direction)) so participants could familiarise themselves with the test requirements. This was followed by a baseline period (30 s of data collection, during which participants were instructed not to move) to bring the haemodynamic status as close to a resting state as possible, and immediately upon completion of this, the participants performed one of the three randomly presented stepping tests.

The fNIRS data were analyzed using Homer2 open-source software in Matlab. The following processing steps were performed in the pipeline: (i) raw data were converted to optical density data (*hmrIntensity2OD*); (ii) active channels were excluded from the analysis if the luminous intensity signal was too weak (<0.01 cd) or too strong (>300 cd), if the signal to noise ratio was too small (mean/standard deviation of the data was <2), and/or if the source–detector separation was < 0 mm or > 45 mm (*hmrR_PruneChannels*); (iii) motion artefacts defined as signal changes greater than pre-specified thresholds (standard deviation threshold = 10; amplitude threshold = 0.3) were removed (*hmrMotionArtifact*); (iv) wavelet transformation of the optical density data was performed to identify motion artefacts (interquartile range = 0.1) (*hmrMotionCorrectWavelet*); (v) data were filtered with a high pass filter at 0.01 Hz and a low pass filter at 0.14 Hz (to remove physiological responses, i.e., heart rate, respiration) (*hmrBandpassFilt*); (vi) the optical density data were converted to [HbO2] and [HHb] (*hmrOD2Conc*); (vii) a correlation-based signal improvement (CBSI) was then employed to reduce noise from head motion [35] (*hmrMotionCorrectCbsi*); (viii) the length of each stepping test data collection was standardised for each individual to the shortest stepping test duration (limited to 60 s); (ix) changes in [HbO2] and [HHb] during each stepping test relative to the preceding baseline period were computed (Δ [HbO2] and Δ [HHb]) and block average data were calculated (*hmrBlockAvg*). For each participant and walking condition, Δ [HbO2] and Δ [HHb] for the DLPFC, SMA and PMC were computed as averages across the relevant channels, as indicated in Figure 2.

### 2.7. Statistical Analysis

Continuous outcome variables were tested for normality of distribution. Outliers were replaced with mean ± 3SD from the averaged data (11 data points representing average activity in a region of interest in a specific test, out of 891 data points(1.2%)). Mixed factorial repeated measures analysis of variance (ANOVA) was used to compare mean Δ [HbO2] and Δ [HHb], as well as mean stepping response times, with concern about falling (low concern about falling vs. high concern about falling) as a between-subject factor and stepping task (CSRT, iCSRT and SST) as a within-subject factor. Statistical significance was set a priori at *p*  <  0.05. Stepping test errors were contrasted between low and high fear groups with a chi-square of Fisher’s exact tests for cross-tabulations. The data were analysed using SPSS v. 24 for Windows (SPSS, Inc., Chicago, IL, USA).

## 3. Results

Participants’ characteristics are presented in Table 1. Of the 99 participants, 28 reported high concern about falling (IconFES score, mean ± SD: 22.2 ± 3.3), and 71 reported low concern about falling (IconFES score, mean ± SD: 13.8 ± 2.6). The groups with high and low concerns about falling were similar with regard to demographics, cognitive performance, and physiological fall risk scores (*p* > 0.05).

### 3.1. Haemodynamics

**Figure 3:**
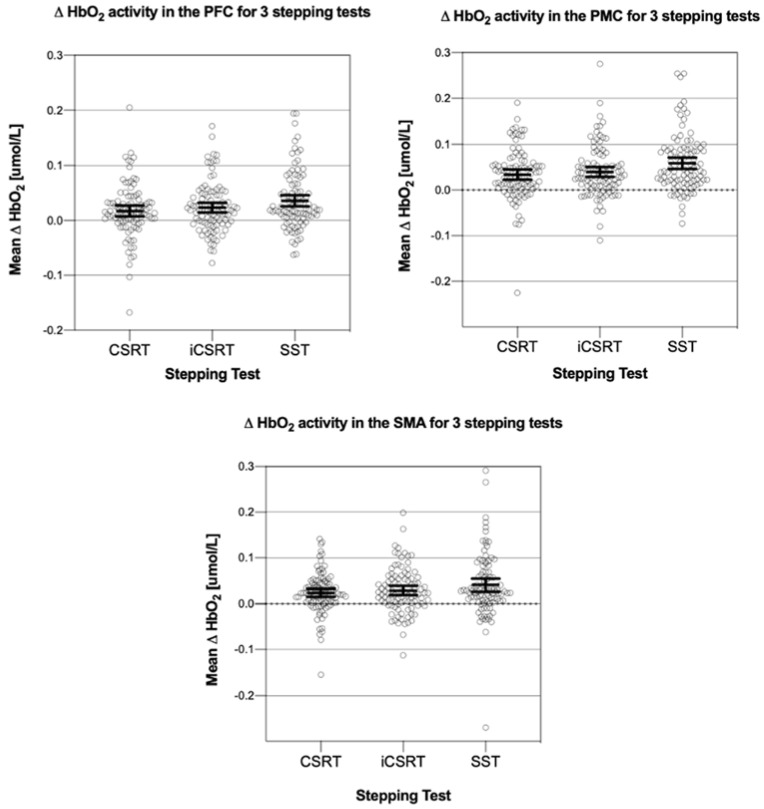
Mean (95%CI) Δ [HbO2] for the stepping tests (choice-stepping reaction time (CSRT); inhibitory CSRT (iCSRT); Stroop stepping test (SST)) in the prefrontal cortex (PFC); premotor cortex (PMC); and supplementary motor area (SMA).

Figure 3 presents the mean Δ [HbO2] levels in the DLPFC, PMC, and SMA during the three stepping tests. Table 2 also presents the findings from statistical analyses. For simplicity, only the HbO2 concentration data are presented in the main body of this paper, with the data for Δ [HHb] presented in Supplementary Material Table S1. Across all conditions, the [HbO2] and [HHb] findings were generally consistent.

There were no significant interactions between concern about falling group and stepping task for Δ [HbO2] or Δ [HHb] in the DLPFC, PMC or SMA. There were also no significant main effects of concern about falling on Δ [HbO2] and Δ [HHb] in the DLPFC, PMC and SMA, indicating that the high concern about falling and low concern about falling groups had similar cortical activity levels in these regions of interest, in each of the three stepping tasks.

There were significant main effects of the stepping task on Δ [HbO2] in the DLPFC, PMC, and SMA. Post-hoc tests revealed that Δ [HbO2] in the SST was significantly higher compared to the CSRT in all regions investigated. Δ [HbO2] in the SST was also significantly higher compared to the iCSRT in the PMC. Post-hoc test results for the Δ [HHb] were in line with those for the Δ [HbO2] (see Table S1).

### 3.2. Stepping Response Times

There was no significant interaction between concern about falling and stepping task for mean stepping response times (F2,188 = 0.257, *p* = 0.774) and no significant main effect of concern about falling on mean stepping response times (F1, 94 = 1.986, *p* = 0.162), indicating that both groups performed similarly in each of the CSRT, iCSRT, and SST tests.

There was a significant main effect of the stepping task on mean stepping response times (F2,188 = 180.253, *p* <0.001). The more complex stepping tests had longer response times, as shown in Table 3. Post-hoc tests revealed a significant difference (95%CI) in mean response times between iCSRT and CSRT of 72 (57 to 87) milliseconds, *p* < 0.001, between SST and CSRT of 305 (254 to 355) milliseconds, *p* < 0.001, and between SST and iCSRT of 233 (185 to 280) milliseconds, *p* < 0.001.

### 3.3. Stepping Errors

There was no significant difference in the proportion of participants who made a stepping error in the CSRT test (χ^2^ = 0.084, *p* = 0.772) or the SST (χ^2^ = 2.465, *p* =0.116) between the low and high concern about falling groups. However, a greater proportion of the high concern about the falling group made a stepping error in the iCSRT test compared with the low concern about the falling group (χ^2^ = 7.514, *p* = 0.006), as shown in Table 4.

## 4. Discussion

This study examined neural efficiency in older community-dwelling people with low and high concern about falling when undertaking three stepping tests of differing complexity—the CSRT, iCSRT, and SST. Step responses were slower, and DLPFC, SMA, and PMC haemodynamic activity levels were higher in the more complex SST compared to the CSRT and iCSRT. However, cortical activity, as well as stepping response times, did not differ significantly between the low and high concern about falling groups across the three stepping tests. The group with high concern about falling made more stepping errors in one of the stepping tests (the iCSRT) compared with the group with low concern about falling.

In contrast to our primary hypothesis, mean Δ [HbO2] and response times did not differ significantly between the groups with low and high concern about falling. These findings also differ from those of Holtzer et al. [15] and St George et al. [16], who reported that fearful older people exhibited neural inefficiency compared to non-fearful older people. This divergence may relate to test differences in that the current stepping tasks while being good proxies for fall risk, may not have induced as much apprehension as a dynamic walking task. In addition, while the participants in the group with high concern about falling met the criterion for this condition based on the validated IconFES tool (total score ≥19), their mean score was 22 out of a total of 40 regarding concern about falling. Therefore, the absence of a notably fearful group possibly contributed to the lack of significant differences in cortical activity and stepping performance between the groups. Further study is needed to investigate the effect of concern about falling on neural efficiency, possibly by inducing fear, e.g., by having participants stand on an elevated platform or priming participants with respect to fear before they complete stepping tasks.

More participants with high concern about falling made stepping errors during the iCSRT task than those with low concern about falling, which suggests that the former group prioritised speed at the expense of accuracy [36]. Compared to the CSRT task, which emphasises the rapid initiation of a step and the SST, which requires a high cognitive load and consistently longer step response times, the iCSRT task requires both response inhibition during no-go trials and selection and rapid initiation of appropriate step movements for the go trials [19]. The higher rate of stepping errors in this task that requires both factors may elucidate why older people are at an increased risk of falls, particularly when undertaking complex daily activities. This finding also aligns with the Attentional Control Theory, which posits that if task demands are too high, mental effort cannot accommodate anxiety-related inefficiencies in working memory. Here, the participants with concern about falling may not have been able to disengage from task-irrelevant stimuli (i.e., ruminating on worrisome thoughts about falling), leading to compromised stepping task performance [11,14].

The finding that cortical activity was significantly higher in all three regions of interest and stepping response times were significantly slower in the SST compared to the CSRT likely reflects that the SST incorporates additional cognitive executive functions, including response inhibition, cognitive flexibility, and increased attention [18]. This finding is in line with the cognitive resource theory, which postulates that brain activity is more pronounced in more demanding tasks [37] and is consistent with the findings of studies that have included walking trials that have involved cognitive tasks and obstacle negotiation [38]. The additional finding that PMC activity was significantly higher during the SST compared with the iCSRT suggests the higher cognitive demand of the SST is especially relevant to the sequencing of movements evoked by external stimuli [24].

Strengths of this study include the large sample size, the use of a validated tool for assessing concern about falling, and the choice of stepping tasks, which have been found to constitute good models for investigating cortical activity patterns and reflect multitasking during balance. The fNIRS data were collected in line with recently published consensus guidelines on fNIRS studies in posture and gait, allowing for reproducible results [39]. In addition, the hypotheses were registered publicly prior to statistical analysis. This study also has limitations. Firstly, in line with the previous studies using this technique [39], the collected fNIRS data had high variability. Three factors may have contributed to this in the current study: (i) We used the fNIRS Optodes’ Location Decider toolbox based on the Brodmann atlas classification [33] to determine the anatomical landmarks for optode placement, as opposed to MRI mapping, which may have reduced the region of interest placement precision; (ii) We used data-based filters at the processing stage which are less effective in removing physiological noise than more recently available short-separation channels and; (iii) Despite using standard procedures to minimise motion artefacts (e. g., wavelet transformation, correlation-based signal improvement) [39], we did not perform additional quality assessments to exclude the presence of bad channels remaining. Secondly, we acknowledge that the reference to the concept of “neural inefficiency”, as used by Holzter and colleagues [15], to characterise a pattern of higher prefrontal cortex activity coinciding with similar or worse walking task performance, in fearful compared to non-fearful older people, should be nuanced. This increased reliance on prefrontal cortical engagement may also reflect compensation for the overburdening of cognitive resources generated by heightened concern about falling. As such, it would be consistent with the compensation-related utilization of neural circuits hypothesis (CRUNCH) [40], which proposes that with increasing task demands, older adults engage additional brain regions to compensate for declining neural structures and functions up to a threshold beyond which such compensatory effects may break down, leading to deactivation in those regions and declining performance. Such a pattern of cortical activity and behavioural performance is illustrated clearly in a functional magnetic resonance imaging study, which required older participants to complete inhibitory tasks of increasing complexity [41]. We, therefore, recommend that future studies investigating the cortical mechanisms underlying concern about falling carefully consider the neural efficiency versus CRUNCH theories. Thirdly, we recognise that to investigate the cortical activity requirements of the cognitive component of the stepping tests in isolation, the use of a simple stepping reaction time test as a “control” condition would be preferred to that of a choice-stepping reaction time test, which includes an inhibitory component [42]. Such an approach would be consistent with that used in cognitive science research [43]. Finally, we concede that postural shifting errors would be more sensitive to discriminate between the groups with and without concern about falling [42], compared to only focusing on stepping error; however, this would require a more sophisticated computerised stepping mat. Our findings that complex stepping tasks require increased cortical input has implications for fall prevention interventions suggesting that cognitive–motor interventions may modulate cortical activity and improve control of inhibitory responses, step planning, and initiation, which may ultimately reduce fall risk [44]. These interventions may be particularly beneficial for older people with high concern about falling for improving appropriate stepping and gait adaptability when negotiating complex, busy environments encountered in everyday life.

## 5. Conclusions

This study’s findings demonstrate that cortical activity is higher in cognitively demanding stepping tasks that require selective attention and inhibition in healthy older people. The finding that the group with high concern about falling did not demonstrate elevated cortical activity levels or reduced stepping performance compared with the group with low concern about falling may reflect the former group’s relatively moderate scores on the IconFES scale.

## Figures and Tables

**Table 1:** Demographics and baseline measures for all participants and among participants with low and high concern about falling. Data are number (%) or mean ± standard deviation.

Variable	Participants (*n* = 99)	Low Concern about Falling (*n* = 71)	High Concern about Falling (*n* = 28)	Mean Difference (95% Confidence Interval)
Age (years)	71.6 ± 4.8	71.1 ± 4.4	73.0 ± 5.5	−1.9 (−4.0, 0.2)
Gender (female)	71 (72%)	50 (70%)	21 (75%)	n/a
Height (cm)	165.6 ± 9.6	165.9 ± 9.7	164.7 ± 9.7	1.3 (−3.0, 5.6)
Weight (cm)	78.0 ± 15.3	77.2 ± 14.4	80.0 ± 17.4	−2.8 (−9.6, 3.9)
Education (years)	15.7 ± 4.4	15.9 ± 4.2	15.1 ± 5.0	0.7 (−1.2, 2.7)
ACER ^1^	95.3 ± 3.8	95.3 ± 4.0	95.1 ± 3.3	0.2 (−1.5, 1.9)
TMT-A (seconds) ^2^	34.3 ± 11.2	32.7 ± 11.0	37.9 ± 11.1	−5.2 (−11.6, 1.3)
TMT-B (seconds) ^3^	77.2 ± 27.5	75.2 ± 26.7	81.7 ± 29.6	−6.5 (−22.7, 9.7)
PPA score ^4^	−0.05 ± 1.12	−0.18 ± 1.08	0.26 ± 1.31	−0.43 (−0.98, 0.13)

^1^ ACER: Addenbrooke’s Cognitive Examination Revised; lower score indicates worse cognitive performance. ^2^ TMT-A: Trail Making Test A; ^3^ TMT-B: Trail Making Test B. Greater time taken indicates worse cognitive performance. ^4^ PPA score: Physiological Profile Assessment Fall Risk Score.

**Table 2:** Δ [HbO2] (μmol/L) in three regions of interest during the three stepping tests for the low and high concern about falling groups. Data are mean ± SD.

Region of Interest	Test	Low Concern about Falling (*n* = 71)	High Concern about Falling (*n* = 28)	Main Effect of Group	Main Effect of Test	Group x Test Interaction
Dorsolateral prefrontal cortex	CSRT ^a^	0.021 ± 0.006	0.009 ± 0.009	F_1, 97_ = 1.124, *p* = 0.292 η^2 ^= 0.011	F_2, 194_ = 5.405, *p* = 0.005 η^2 ^= 0.053	F_2, 194_ = 0.175, *p* = 0.840 η^2 ^= 0.002
	iCSRT	0.026 ± 0.005	0.017 ± 0.009			
	SST ^a^	0.037 ± 0.006	0.032 ± 0.010			
Premotor cortex	CSRT ^b^	0.036 ± 0.007	0.029 ± 0.011	F_1, 97_ = 0.545, *p* = 0.462 η^2 ^= 0.006	F_2, 194_ = 7.348, *p* = 0.001 η^2 ^= 0.070	F_2, 194_ = 0.039, *p* = 0.961 η^2 ^< 0.001
	iCSRT ^b^	0.043 ± 0.007	0.033 ± 0.011			
	SST ^b^	0.061 ± 0.007	0.054 ± 0.012			
Supplementary motor area	CSRT ^c^	0.023 ± 0.005	0.025 ± 0.008	F_1, 97_ = 0.043, *p* = 0.837 η^2 ^< 0.001	F_2, 194_ = 4.253, *p* = 0.016 η^2 ^= 0.042	F_2, 194_ = 0.198, *p* = 0.820 η^2 ^= 0.002
	iCSRT	0.030 ± 0.006	0.026 ± 0.009			
	SST ^c^	0.042 ± 0.006	0.038 ± 0.006			

CSRT: Choice Stepping Reaction Time; iCSRT: Inhibitory Choice Stepping Reaction Time; SST: Stroop stepping test. ^a^ Significant post-hoc test; CSRT versus SST, *p* = 0.008. ^b^ Significant post-hoc tests; CSRT versus SST, *p* = 0.001; iCSRT versus SST, *p* = 0.014. ^c^ Significant post-hoc test; CSRT versus SST, *p*= 0.010. Effect sizes computed with Partial Eta Squared: η^2^= 0.01 indicates a small effect; η^2^ = 0.06 indicates a medium effect; and η2 = 0.14 indicates a large effect.

**Table 3:** Stepping response times (milliseconds) in the three stepping tests for the low and high concern about falling groups. Data are mean ± SD.

Test	Low Concern About Falling (*n* = 71)	High Concern About Falling (*n* = 28)	Main Effect of Group	Main Effect of Test	Group x Test Interaction
**CSRT**	777 ± 10	816 ± 17	F_1, 94_ = 1.986, *p* = 0.162 η^2 ^= 0.021	F_2, 188_ = 180.253, *p* <0.001 η^2 ^= 0.657	F_2, 188_ = 0.257, *p* = 0.774 η^2 ^= 0.003
**iCSRT**	858 ± 12	880 ± 19			
**SST**	1079 ± 24	1124 ± 39			

CSRT: Choice Stepping Reaction Time; iCSRT: Inhibitory Choice Stepping Reaction Time; SST: Stroop stepping test effect sizes computed with Partial Eta Squared: η2= 0.01 indicates a small effect; η2 = 0.06 indicates a medium effect; and η2 = 0.14 indicates a large effect.

**Table 4:** Number of participants who made no errors versus 1 or more errors in the three stepping tests.

	Low Concern about Falling	High Concern about Falling	Total
**CSRT**			
No error	25	9	34
1 or more errors	46	19	65
Total	71	28	99
**iCSRT**			
No error	42	8	50
1 or more errors ^a^	29	20	49
Total	71	28	99
**SST**			
No error	47	23	70
1 or more errors	24	5	29
Total	71	28	99

CSRT: Choice Stepping Reaction Time; iCSRT: Inhibitory Choice Stepping Reaction Time; SST: Stroop stepping test. ^a^ Greater proportion of participants with high concern about falling who made an error in the iCSRT compared to participants with low concern about falling (*p* < 0.05).

## Data Availability

The data are not publicly available due to ethical restrictions.

## Supplementary Materials

The following supporting information can be downloaded at https://www.mdpi.com/article/10.3390/brainsci13121675/s1, Table S1: Δ [HHb] (μmol/L) in three regions of interest during the three stepping tests for the low and high concern about falling groups. Data are mean ± SD.
