# Supplementary material for: Prefrontal and Motor Planning Cortical Activity during Stepping Tasks Is Related to Task Complexity but Not Concern about Falling in Older People: A fNIRS Study
Source: Brain Sci. 2023 Dec 5;13(12):1675. doi: 10.3390/brainsci13121675 (PMC10742256; doi:10.3390/brainsci13121675)
Supplement: Supplementary file 1 [file brainsci-13-01675-s001.zip › brainsci-2664692-supplementary.pdf]

Table S1: Δ [HHb] (μmol/L) in three regions of interest during the three stepping tests for the low and high concern about falling groups. Data presented are mean ± SD

**Region of interest**	**Test**	**Low concern about falling (n=71)**	**High concern about falling (n=28)**	**Main effect of group**	**Main effect of test**	**Group x test interaction**
Prefrontal cortex	CSRT [a]	-0.007 ± 0.018	-0.004 ± 0.016	$F_{1,95}$=0.195, p=0.660	**$F_{2,190}$=3.618, p=0.029**	$F_{2,190}$=0.705, p=0.495
	iCSRT [a]	-0.008 ± 0.016	-0.005 ± 0.016			
	SST [a]	-0.010 ± 0.016	-0.012± 0.020			
Pre-motor cortex	CSRT [b]	-0.015 ± -0.021	-0.013 ± 0.021	$F_{1,95}$=0.231, p=0.632	**$F_{2,190}$=7.447, p<0.001**	$F_{2,190}$=0.044, p=0.957
	iCSRT [b]	-0.016 ± 0.025	-0.013 ± 0.021			
	SST [b]	-0.024 ± 0.027	-0.022 ± 0.026			
Supplementary motor area	CSRT [c]	-0.008 ± -0.013	-0.008 ± 0.018	$F_{1,95}$=0.047, p=0.829	**$F_{2,190}$=3.109, p=0.047**	$F_{2,190}$=0.149, p=0.862
	iCSRT	-0.010 ± 0.018	-0.009 ± 0.017			
	SST [c]	-0.014 ± 0.021	-0.012 ± 0.024			

CSRT: Choice Stepping Reaction Time; iCSRT: Inhibitory Choice Stepping Reaction Time; SST: Stroop Stepping Test

[a] significant post-hoc tests, CSRT versus SST, p=0.019; iCSRT versus SST, p=0.038.

[b] significant post-hoc tests, CSRT versus SST, p=0.001; iCSRT versus SST, p=0.002.

[c] significant post-hoc test, CSRT versus SST, p= 0.027.
